# Evaluation of call volume and negative emotions in emergency response system telecommunicators: a prospective, intensive longitudinal investigation

**DOI:** 10.3934/publichealth.2022027

**Published:** 2022-03-28

**Authors:** Matthew Hoang, Elizabeth Hillier, Chris Conger, Devan N. Gengler, Cody W. Welty, Candace Mayer, Patricia L. Haynes

**Affiliations:** 1 Department of Physiology, College of Medicine, University of Arizona, Tucson, AZ, USA; 2 Health Promotion Sciences, Mel and Enid Zuckerman College of Public Health, University of Arizona, Tucson, AZ, USA; 3 Tucson Fire Department, City of Tucson, Tucson, AZ, USA

**Keywords:** emergency medical service, telecommunication, emergency dispatchers, emotion, irritable mood, psychological stresses, exposure, occupational

## Abstract

Emergency telecommunicators are essential first responders tasked with coordinated communication within the emergency response system (ERS). Despite their exposure to significant job demands, little is known about the effect of call load or call type on emotional state within these workers. Therefore, we employed a prospective, intensive longitudinal design to examine whether emergency-eligible call volume would lead to higher intensity negative emotions post-shift when controlling for pre-shift negative emotions and a number of other work and individual factors, including work duration and night shift. A total of 47 ERS telecommunicators (dispatchers, operators, other) completed ratings over working shifts within a two-week period. Call frequency was gathered through the agency Computer-Assisted Dispatch database. Negative emotions of irritation, stress, worry, and fatigue were measured through the Visual Analogue Scale administered before and after shift. Mixed linear modeling demonstrated that telecommunicators who received more calls per hour (Estimate = 3.56, SE = 1.44, p < 0.05) and more-than-usual calls per hour (Estimate = 1.97, SE = .94, p < 0.05) had higher levels of post-shift irritability. Longer-than-usual working hours also predicted higher levels of post-shift irritability (Estimate = 1.32, SE = 0.59, p < 0.05). Call volume did not predict other negative emotions, although secondary analyses demonstrated that a larger number of chronic calls lead to greater levels of post-shift worry. ERS telecommunication agencies aiming to reduce negative emotions in workers may benefit from implementing policies and programs that target working hours, call load, and work-life balance.

## 1. Introduction

The 911 emergency response system (ERS) is a vital public health safety net that handles approximately 199.3 million calls per year in the United States (U.S.). Over the last 50 years, the ERS in the U.S. has grown tremendously, with call volume increasing by approximately 18.4 million calls from two years prior in 2018 [1]. Emergency response system telecommunicators are the “first of the first responders” who receive these ERS calls, interface directly with the public, and dispatch calls as part of a coordinated response system. These workers are faced with time-sensitive choices about how to solve complex problems while managing the strict requirements of workplace presence. Emergency telecommunication is a career characterized by high demands with low autonomy, making ERS telecommunication a highly stressful career choice.

Few studies have examined stress exposure in ERS telecommunicators. Only nine quantitative studies were identified in a recent systematic review examining the psychological health of emergency dispatch operatives [2]. The majority of the studies reviewed employed a cross-sectional design and were rated as overall weak quality (5 of the 9), potentially due to limited power. Despite this, results from this review demonstrated that workers within this industry are vulnerable to stress reactions. Prior to the current Covid-19 global pandemic, research indicated that 18% of ERS telecommunicators screened positive for posttraumatic stress disorder (PTSD) [3], and 15% screened positive for major depressive disorder [4]. Rates in ERS telecommunicators were substantially higher than the general population at that time, when 5% of individuals in the U.S. screened positive for PTSD [5] and 8% screened positive for depression [6].

Although ERS telecommunicators are not present at the scenes of distress, they are often exposed to potentially psychologically traumatic events [7], such as giving cardio-pulmonary resuscitation (CPR) instructions, calming a child while an intruder is in their home, and helping survivors of a fatal car crash [7]. A report from the Center for American Progress analyzed open data from 911 calls from eight U.S. cities and discovered that 18–34% of calls were life-threatening emergencies, 23–38% of calls were nonurgent, and 34–42% of calls were medium-priority, non-life threatening incidents [8]. Non-emergency calls can strain the ERS leading to an ineffective use of scarce resources and time that could be utilized for solving a life-threatening issue [9].

In addition to mental health disorders, approximately 14.5% of ERS telecommunicators report symptoms of compassion fatigue (CF, [10]). Compassion fatigue describes the state of tension resulting from secondary exposure (i.e., exposures of patients experiencing trauma) of trauma [11]. It leads to alcohol and drug abuse, decreased ability to feel sympathy and empathy, and a reduced ability to make decisions for patients/clients [12]. A key symptom of compassion fatigue is irritability, a derivative of anger [13],[14], which is also experienced by ERS workers [4]. Irritability is also a symptom of major depressive disorder and PTSD. Higher levels of irritability are associated with increased risk of suicide, decreased quality of life and increased stressful interpersonal events [15]–[18]. Research has demonstrated that irritable workers may be less able to tolerate frustrating work stressors and must therefore put forth more effort to overcome stressors [19].

Unfortunately, very little is known about irritability and other emotional disturbances in ERS telecommunicators and how negative emotions relate to job demands. The purpose of this study is to examine whether ERS telecommunicators who take more dispatched calls (i.e., calls that are eligible for emergency response) have higher levels of post-shift irritability, as compared to ERS telecommunicators who take fewer dispatched calls, when controlling for pre-shift irritability, shift duration, night shift work, and other relevant covariates. We also tested whether changes in irritability are consistent with changes in other negative emotions, including feelings of stress, worry, and fatigue.

## 2. Materials and methods

### 2.1. Participants and procedures

This prospective, intensive longitudinal study was approved by the University of Arizona Human Subjects Protection Program. Participation was entirely voluntary. Written informed consent was provided by 47 full-time employees in the job position of ERS telecommunicator. All employees worked in one agency that serves a mid-sized metropolitan area in the Southwest region of the U.S. between the months of June–January. Eligible individuals were English-fluent between the ages of 18–65 years. Individuals in all positions were eligible to participate, as long as they answered emergency calls. The majority of the sample consisted of participants employed as dispatchers. Dispatchers are tasked with receiving information about the call and relaying that information to first responders. Operators are ERS telecommunicators who interface with the caller. At this agency, all positions, including supervisors, interface with the caller to ensure adequate coverage. Recruitment occurred through study flyers and emails administered to the entire agency. Individuals were excluded from participation if they did not attend more than 7 days of work during the study period. Cases were excluded if participants did not receive calls at work that day or did not complete a post-shift emotion rating on the Visual Analogue Scale (VAS), the primary outcome assessment (see below).

At the initial visit, study staff informed participants about the study purpose and obtained written informed consent to participate. A battery of measures was administered to describe the sample and assess for potential covariates (see Measures below). Participants were then instructed to complete the emotion rating when on-shift over the 14 days: twice per day, within the first hour and last hour of their shift. The frequency of calls was retrieved from the computer assisted dispatch (CAD) database maintained by the agency, following in-person data collection.

### 2.2. Measures

All assessments in this study were completed through Research Electronic Database Capture (REDCap) or paper, if the participant preferred paper or experienced internet failure. All paper records were subject to independent data double-entry and reconciliation. A demographic, medical, and occupational history interview was administered at the baseline visit to describe the sample.

The dependent variables were measured by the VAS [20],[21], a self-report, valid and reliable measure developed for the daily assessment of various emotions. Participants were asked to place a single mark on a 100 millimeters line to best illustrate how they were currently feeling as described by anchors on the end of each line. The mark is measured in millimeters and scores range from 0–100. The following negative emotions were tested: irritated, stressed, worried, and fatigued.

Shift demands for each participant were captured from the agency's Computer Assisted Dispatch (CAD) system log that code the following characteristics about the call including: telecommunicator identity, date, number of calls and event type. Calls are entered into the CAD database when they are determined eligible for emergency response and routed for dispatching. Nonemergency calls are not entered into the CAD database. An expert consensus panel of four experienced supervisors participated in categorizing call types as acute or chronic. Acute calls were considered challenging and required active intervention by the ERS telecommunicator (e.g., childbirth delivery, severe injury or fatality, choking, swift water rescue). Chronic calls were attributed to social services issues and might involve negative interpersonal exchanges (e.g., suspicious person or vehicle, loud party, impaired/intoxication, or patient feeling sick).

### 2.3. Data analyses

The current dataset was imbalanced since employees worked a mixed number of shifts over the 14 days period. Missingness was explored through the creation of two variables, one at the level of case and the other at the level of individual, to assess the missingness at random (MAR) assumption. Several theoretically plausible missingness scenarios were assessed through a series of univariate analyses examining whether missingness was associated with observed values of pre-shift emotion variables, day of assessment, number of CAD calls, work duration, night shift, and a number of person-level covariates (length of time employed at the organization, length of time employed in the position, age, gender). Descriptive statistics were employed to describe the sample.

Covariates were chosen for testing based on their prior documented associations with negative emotional state and job strain: night shift, time (days in the study), age (grand mean centered), gender, and under-represented racial/ethnic minority status (1 = not white or Hispanic; 0 = white or not Hispanic). Significant relationships between these fixed variables and the relevant dependent variable were included in the final models. In addition, final models were analyzed with time in the model to ensure the proposed relationships were retained when controlling for time.

In all models, the corresponding pre-shift emotion was included as an independent variable, along with shift duration (within and between subject effects) and the mean number of dispatched calls per hour (within and between subject effects). For secondary analyses, the mean number of dispatched calls per hour was separately replaced with (a) the mean number of acute calls per hour and (b) mean number of chronic calls per hour. All continuous independent variables were disaggregated into between- and within-person effects by computing the person-specific mean (between-person effect) and subtracting the person-specific mean from the individual's time specific value (within-person effect) [22]. All between-person effects were grand mean centered. Four dependent variables were tested separately and included VAS scores post-shift: irritated, stressed, worried, fatigued.

A preliminary null model was employed to disaggregate between within and between subject variability and confirm the employment of mixed effects linear modeling. Mixed effects linear modeling was employed using the mixed procedure in IBM SPSS 27.0 with a repeated statement to designate a first-order autoregressive covariance matrix based on time (study day). The participant intercept was also included in all models as a random effect with a compound symmetry covariance structure. All models were fit using restricted maximum likelihood estimation.

## 3. Results

### 3.1. Preliminary analyses

Participants completed the VAS at post-shift 85% of days they received CAD calls. Missing VAS post-shift scores were associated with an increased likelihood of working night shift, χ^2^ (1, n = 330) = 5.37, p = 0.02, supporting the inclusion of this covariate in all models. Approximately 49% (n = 24) of individuals were missing at least one post-shift VAS outcome; no individual-level factors were associated with missingness. The final imbalanced sample consisted of 281 data points. No variables contained 5% or more missing cases. Only pre-shift VAS irritability contained missing values (n = 12/281, 4.3%). The low proportion of missing data among observed values and inclusion of night shift work in the model supports the credibility of the Missingness at Random (MAR) assumption without correction for missingness.

**Table 1.** Participant characteristics in mean (SD) or percentage.

Characteristic	M	SD
Age	37.87	9.85
Gender, female	72%	
Under-represented minority	38%	
Mo. employed at organization	78.23	81.56
Mo. employed in current position	56.56	53.60
Job Position		
Dispatcher	55%	
Operator	30%	
Other	23%	
No. days on-shift in study period	5.98	2.27
Shift duration, hrs	11.08	1.38
No. night-shifts worked	0.32	0.89
CAD call frequency		
No. CAD calls / hr	2.17	1.34
No. CAD calls / hr, acute	0.02	0.03
No. CAD calls / hr, chronic	0.42	0.65
Negative Emotion		
Irritated, pre-shift	32.73	17.60
Irritated, post-shift	37.62	19.23
Stressed, pre-shift	34.77	19.00
Stressed, post-shift	40.20	19.12
Worried, pre-shift	31.12	16.15
Worried, post-shift	32.78	17.40
Fatigued, pre-shift	44.26	16.52
Fatigued, post-shift	56.20	17.00

*Note: CAD = Computer-Assisted Dispatch system. SD = standard deviation. All emotion ratings were conducted by Visual Analogue Scale ranging from 0 to 100. n = 47.

See Table 1 for person-level means and standard deviations, and Supplementary Table 1 for correlations of within-subject study variables. Supplementary Table 2 demonstrates findings from the null models that tests the disaggregation of between- and within-subject variance components. The intraclass correlation coefficients (between-person level variance/total variance) demonstrated adequate clustering to support the use mixed linear modeling. Daily changes in negative emotions within individuals were responsible for 46–57% of the total variance.

The only covariate to emerge as statistically significant was time when predicting post-shift feelings of worry. Therefore, this covariate was included in each of the final models for this outcome.

**Table 2.** Results of multilevel models examining post-shift negative emotion from the number of dispatched calls taken by ERS telecommunicators.

Fixed Effects	Irritable		Stressed		Worried		Fatigued	
	Estimate	SE	Estimate	SE	Estimate	SE	Estimate	SE
Intercept	–37.04	21.52	–15.15	20.32	–20.06	14.75	–46.65*	22.08
Night shift worked	–2.13	6.70	–0.83	5.98	3.09	5.22	7.32	6.29
Pre-shift emotion, BS	0.87***	0.11	0.82***	0.10	0.91***	0.08	0.65***	0.12
Pre-shift emotion, WS	0.05	0.08	0.04	0.07	0.19**	0.06	0.22**	0.06
Work duration, BS	3.33	1.68	2.03	1.60	2.37*	1.13	6.91***	1.70
Work duration, WS	1.32*	0.59	1.34*	0.53	0.60	0.47	0.71	0.56
No. calls per hour, BS	3.56*	1.44	1.85	1.39	0.07	0.98	2.41	1.47
No. calls per hour, WS	1.97*	0.94	0.89	0.84	–0.18	0.75	–0.35	0.88
Time, days					0.39	0.25		

*Note: WS = within subject effects. BS = between subject effects. SE = Standard Error. Between subject values are grand mean centered. Within subject variables are person centered, except for night-shift and time. All pre-shift emotions correspond to the same post-shift emotion. *p < 0.05, **p < 0.01, ***p < 0.001.

### 3.2. Main analyses

The results from each of the final main emotion models are reported in Table 2. Across all models, main effects demonstrated that higher levels of post-shift negative emotion were associated with higher between subject levels of the same pre-shift negative emotion. In addition, higher levels of post-shift worry and fatigue were associated with higher within subject levels of the same pre-shift emotion.

Work duration was relevant for post-shift negative emotions. Individuals working longer shifts than their coworkers were more likely to feel worried and fatigued post-shift. In addition, shifts that were longer than a person's usual (e.g., overtime) were associated with higher levels of post-shift irritability and feelings of stress.

Overall call load was only statistically significant for post-shift irritability. Both between and within subject main effects demonstrated that higher levels of post-shift irritability were associated with both (a) more emergency calls per hour than other ERS telecommunicators and (b) more emergency calls per hour than a person's average number of calls.

Next, we repeated analyses separately substituting the between and within effects for the number of calls per hour with within and between subject variables indicating (a) the number of acute calls per hour and (b) the number of chronic calls per hour. In all four negative emotion models, neither the within nor the between subject acute call variables were predictive of post shift emotions when adjusting for other covariates in the model (See Table 3). Overall results remained consistent for chronic calls. However, a between subject effect emerged indicating that more chronic calls were associated with greater levels of post-shift worry (see Supplementary Table 4). The within-subject effect for the number of chronic calls was not significant.

**Table 3.** Results of multilevel models examining post-shift negative emotion from the number of dispatched acute calls taken by ERS telecommunicators.

Fixed Effects	Irritable		Stressed		Worried		Fatigued	
	Estimate	SE	Estimate	SE	Estimate	SE	Estimate	SE
Intercept	–12.79	20.00	–1.15	18.03	–21.02	12.56	–28.21	19.79
Night shift worked	–2.82	6.81	–0.41	5.96	2.78	5.17	8.33	6.28
Pre-shift emotion, BS	0.83***	0.12	0.79***	0.10	0.92***	0.07	0.63***	0.12
Pre-shift emotion, WS	0.05	0.08	0.04	0.07	0.18**	0.06	0.22**	0.06
Work duration, BS	1.87	1.69	1.19	1.54	2.50*	1.04	5.89**	1.65
Work duration, WS	0.92	0.57	1.17*	0.50	0.68	0.45	0.76	0.53
No. acute calls per hour, BS	–7.24	75.73	27.92	69.39	–49.36	46.54	–4.26	73.80
No. acute calls per hour, WS	–13.49	31.77	–36.04	27.76	–29.20	25.09	–8.97	29.25
Time, days					0.39	0.25		

*Note: WS = within subject effects. BS = between subject effects. SE = standard error. Between subject values are grand mean centered. Within subject variables are person centered, except for night-shift and time. All pre-shift emotions correspond to the same post-shift emotion. *p < 0.05, **p < 0.01, ***p < 0.001.

**Table 4.** Results of multilevel models examining post-shift negative emotion from the number of chronic dispatched calls taken by ERS telecommunicators.

Fixed Effects	Irritable		Stressed		Worried		Fatigued	
	Estimate	SE	Estimate	SE	Estimate	SE	Estimate	SE
Intercept	–26.11	20.79	–14.22	17.93	–29.77*	12.99	–31.81	21.60
Night shift worked	–3.30	6.78	–1.66	5.94	2.53	5.12	8.81	6.25
Pre-shift emotion, BS	0.89***	0.12	0.83***	0.09	0.94***	0.07	0.64***	0.13
Pre-shift emotion, WS	0.05	0.08	0.04	0.07	0.18**	0.06	0.21**	0.06
Work duration, BS	2.67	1.69	2.01	1.48	2.98**	1.04	6.15**	1.70
Work duration, WS	0.93	0.60	1.24*	0.53	0.72	0.46	1.01	0.55
No. chronic calls per hour, BS	5.35	3.14	5.27	2.70	4.20*	1.89	1.46	3.24
No. chronic calls per hour, WS	–0.55	3.52	0.49	3.10	–0.02	2.75	4.72	3.22
Time, days					0.41	0.25		

*Note: WS = within subject effects. BS = between subject effects. SE = standard error. Between subject values are grand mean centered. Within subject variables are person centered, except for night-shift and time. All pre-shift emotions correspond to the same post-shift emotion. *p < 0.05, **p < 0.01, ***p < 0.001.

## 4. Discussion

This study examined whether ERS telecommunicators who received more dispatched calls have higher levels of post-shift irritability and other negative emotions, as compared to ERS telecommunicators who receive fewer dispatched calls. The results demonstrated that ERS telecommunicators experienced more post-shift irritability when they 1) receive more emergency calls compared to their coworkers, 2) receive more emergency calls compared to their usual number of calls, 3) worked longer shifts compared to their usual, and 4) started work with higher pre-shift irritability. As expected, higher levels of pre-shift negative emotions were the most consistent predictor of post-shift negative emotion. Taken together, these results support that burnout and other conditions associated with irritability at work are a result of excessive job demands, shift length, and home recovery of mood [23].

These results are consistent with previous research reporting that ERS telecommunicators experience significant amounts of stress due to call load [3],[5]. According to the Job Demand-Control (JDC) model, the combination of low autonomy and high demand on the job is associated with higher mental strain and reduced wellbeing [24]. On average, ERS telecommunicators in this study received 2.17 dispatched calls per hour. This may seem low because dispatched calls represent the minority of calls that ERS telecommunicators receive, as confirmed through internal evaluation at this agency [23]. Although all employees in this study take emergency-eligible calls as needed, interfacing with the public is the primary responsibility of telecommunicators in the position of operator. Because the overall number of calls appears to predict irritability, this suggests that individuals in the position of operator may be particularly vulnerable to irritability; however, further work with a larger sample is necessary to test this finding more specifically. In addition to the total number of calls, the within-subject effect contributed by our results suggest that work exceeding an individual's typical average call volume contributes to feelings of post-shift irritability.

Of all the negative emotions, the total number of dispatched calls was only associated with irritability. These results are compelling given previous research demonstrating that both state and trait anger are predictive of psychopathology in ERS telecommunicators [3]. Other healthcare workers experience high rates of irritability as well [25]. Given the negative effect of irritability on health [26] and the high prevalence of irritability in the workplace [27], irritability may be an important target for future disease prevention in the workplace.

Surprisingly, acute call volume had no effect on post-shift negative emotion. The only witnessed effect for call type was for chronic calls. More chronic calls led to greater levels of post-shift worry. The impact of chronic calls on negative emotions may be a result of their function as hindrance stressors. Hindrance stressors are workplace stressors that are barriers to personal achievement, such as job ambiguity, organizational politics, and red tape. Hindrance stressors have previously been associated with insomnia [28]. ERS telecommunicators are trained for emergency response. Significant time spent responding to chronic, nonurgent calls may create role ambiguity for these workers, leading to negative emotion states. This interpretation is supported by qualitative research in firefighters who express frustration responding to a high volume of low acuity medical calls that are not emergencies and instead social service situations [9].

Greater overall levels of negative emotions pre-shift were the strongest predictors of negative emotions post-shift, suggesting that dispositional or stable individual factors are important determinants of all negative emotions. In addition, greater-than-usual feelings of worry and fatigue at the beginning of shift led to increased levels of worry and fatigue post-shift. Thus, the data suggest that home life activities occurring prior to shift could play a major role in experiencing worry and fatigue at the end of the shift. Recovery is a process that allows people to rest and replenish their resources needed for their next day of work. However, recovery may not always be feasible depending upon working times and may be especially difficult for employees who face active home demands during off-work times [29]. This finding supports the active promotion of work-life balance interventions that promote rest and recovery in ERS telecommunicators, such as sleep health promotion [30].

Lastly, results demonstrated that individuals working longer shifts were more likely to experience worry and fatigue, which is consistent with prior research demonstrating a significant relationship between longer shifts and negative emotions, including irritability, fatigue, anxiety, and depression [31],[32]. Longer working hours have been associated with cardiovascular disease, mental health issues, alcoholism and sleep disturbances [33],[34]. Interestingly, the total number of hours worked was not associated with feelings of irritability and stress; stress and irritability were most affected by work hours that were higher than their own usual (within-subject effect). Prior research demonstrates that individuals vary greatly in their ability to maintain high work performance standards under different work durations [35]. Taken together, these findings support that post-shift emotions of stress and irritability may be sensitive indicators of excessive job demand and overwork, as compared to feelings of worry and fatigue, which may be more relevant at the individual level. Further research is necessary to specifically test this hypothesis.

Strengths of this study include the intensive longitudinal design that examined day-to-day prospective relationships before and after shifts and an objective assessment of call data. Limitations of this study were the small sample size and short time scale. In addition, the current analyses did not take non-emergency call load into account. Similar to chronic calls, non-emergency calls can be conceptualized as hindrance stressors that may potentiate negative emotional response. Further research is warranted to examine the long-term mental health of ERS telecommunicators. Few studies have investigated these essential first responders. Also, few studies have examined irritability or anger within the context of workplace wellness. Most occupational research has examined irritability through the lens of burnout and compassion fatigue, a marker of worker performance. Irritability is especially important to examine given the well-documented effect of anger on cardiovascular disease, which is the leading cause of death in other first responder groups, including police [36] and firefighters [37].

## 5. Conclusions

Findings from this study highlight numerous factors associated with work design and work-life balance that are associated with post-shift emotion. Future studies may benefit from the testing of work design interventions that distribute emergency call volume load among ERS telecommunicators, as well as interventions that reduce shift length. In addition, workplaces may benefit from mental health promotion strategies targeting both pre-shift and post-shift negative emotions. Lastly, research is needed that examines positive emotions across shift and factors that confer resiliency to stress in first responders. Taken together, these findings support and extend research demonstrating that ERS telecommunicators are exposed to significant work stressors that confer vulnerability to negative emotion states. This work is highly relevant to public health preparedness, as the health of ERS telecommunicators directly affects the efficiency of community response.

## References

[1] National 911 Annual Report 2019 Data (2020). From: National 911 Program - Office of Emergency Medical Services, National Highway Traffic Safety Administration, U.S..

[2] Golding SE, Horsfield C, Davies A (2017). Exploring the psychological health of emergency dispatch centre operatives: a systematic review and narrative synthesis. PeerJ.

[3] Lilly MM, Allen CE (2015). Psychological inflexibility and psychopathology in 9-1-1 telecommunicators. J Trauma Stress.

[4] Pierce H, Lilly MM (2012). Duty-related trauma exposure in 911 telecommunicators: considering the risk for posttraumatic stress. J Trauma Stress.

[5] Goldstein RB, Smith SM, Chou SP (2016). The epidemiology of DSM-5 posttraumatic stress disorder in the United States: results from the National Epidemiologic Survey on Alcohol and Related Conditions-III. Soc Psychiatry Psychiatr Epidemiol.

[6] Brody DJ, Pratt LA, Hughes JP (2018). Prevalence of depression among adults aged 20 and over: United States, 2013–2016. NCHS Data Brief.

[7] Meischke H, Painter I, Lilly M (2015). An xxploration of sources, symptoms and buffers of occupational stress in 9-1-1 emergency call centers. Ann Emerg Dispatch Response.

[8] Irwin A, Pearl B (2020). The Community Responder Model: How Cities Can Send the Right Responder to Every 911 Call, Center for American Progress.

[9] Cannuscio CC, Davis AL, Kermis AD (2016). A strained 9-1-1 system and threats to public health. J Community Health.

[10] Goold M (2011). Compassion fatigue, compassion satisfaction, burnout, and peritraumatic disassociation in 9-1-1 telecommunicators; 9-1-1 in crisis.

[11] Figley CR (1995). Compassion fatigue: Coping with secondary traumatic stress disorder in those who treat the traumatized.

[12] Pfifferling JH, Gilley K (2000). Overcoming compassion fatigue. Fam Pract Manag.

[13] Stringaris A, Taylor E (2015). Disruptive mood: Irritability in children and adolescents.

[14] Toohey MJ, Digiuseppe R (2017). Defining and measuring irritability: Construct clarification and differentiation. Clin Psychol Rev.

[15] Fava M, Hwang I, Rush AJ (2010). The importance of irritability as a symptom of major depressive disorder: results from the National Comorbidity Survey Replication. Mol Psychiatry.

[16] Perlis RH, Fava M, Trivedi MH (2009). Irritability is associated with anxiety and greater severity, but not bipolar spectrum features, in major depressive disorder. Acta Psychiatr Scand.

[17] Pickles A, Aglan A, Collishaw S (2010). Predictors of suicidality across the life span: The Isle of Wight study. Psychol Med.

[18] Sahl JC, Cohen LH, Dasch KB (2009). Hostility, interpersonal competence, and daily dependent stress: A daily model of stress generation. Cognit Ther Res.

[19] Fida R, Paciello M, Barbaranelli C (2014). The role of irritability in the relation between job stressors, emotional reactivity, and counterproductive work behaviour. Eur J Work Organ Psychol.

[20] Marsh-Richard DM, Hatzis ES, Mathias CW (2009). Adaptive Visual Analog Scales (AVAS): a modifiable software program for the creation, administration, and scoring of visual analog scales. Behav Res Methods.

[21] Aitken RC (1969). Measurement of feelings using visual analogue scales. Proc R Soc Med.

[22] Raudenbush SW, Bryk AS (2002). Hierarchical linear models: Applications and data analysis methods.

[23] Trachik B, Marks M, Bowers C (2015). Is dispatching to a traffic accident as stressful as being in one? Acute stress disorder, secondary traumatic stress, and occupational burnout in 911 emergency dispatchers. Ann Emerg Dispatch Response.

[24] Karasek RA (1979). Job demands, job decision latitude, and mental strain: Implications for job redesign. Admin Sci Quart.

[25] Boran A, Shawaheen M, Khader Y (2012). Work-related stress among health professionals in northern Jordan. Occup Med.

[26] Dougherty DD, Rauch SL, Deckersbach T (2004). Ventromedial prefrontal cortex and amygdala dysfunction during an anger induction positron emission tomography study in patients with major depressive disorder with anger attacks. Arch Gen Psychiatry.

[27] Posternak MA, Zimmerman M (2002). Anger and aggression in psychiatric outpatients. J Clin Psychiatry.

[28] Haynes PL, Wolf RL, Howe GW (2021). Unemployed individuals reporting hindrance work stress at previous job have increased likelihood of insomnia disorder. Int J Behav Med.

[29] Zijlstra FRH, Sonnentag S (2006). After work is done: Psychological perspectives on recovery from work. Eur J Work Organ Psychol.

[30] Crain TL, Hammer LB, Bodner T (2019). Sustaining sleep: Results from the randomized controlled work, family, and health study. J Occup Health Psychol.

[31] Kikuchi H, Odagiri Y, Ohya Y (2020). Association of overtime work hours with various stress responses in 59,021 Japanese workers: Retrospective cross-sectional study. PLoS One.

[32] Park S, Kook H, Seok H (2020). The negative impact of long working hours on mental health in young Korean workers. PLoS One.

[33] Virtanen M, Ferrie JE, Gimeno D (2009). Long working hours and sleep disturbances: the Whitehall II prospective cohort study. Sleep.

[34] Virtanen M, Jokela M, Nyberg ST (2015). Long working hours and alcohol use: systematic review and meta-analysis of published studies and unpublished individual participant data. BMJ.

[35] Van Dongen HP, Belenky G (2009). Individual differences in vulnerability to sleep loss in the work environment. Ind Health.

[36] Soteriades ES, Smith DL, Tsismenakis AJ (2011). Cardiovascular disease in US firefighters: a systematic review. Cardiol Rev.

[37] Franke WD, Ramey SL, Shelley MC (2002). Relationship between cardiovascular disease morbidity, risk factors, and stress in a law enforcement cohort. J Occup Environ Med.

