# Supplementary material for: Evaluation of call volume and negative emotions in emergency response system telecommunicators: a prospective, intensive longitudinal investigation
Source: AIMS Public Health. 2022 Mar 28;9(2):403–14. doi: 10.3934/publichealth.2022027 (PMC9114781; doi:10.3934/publichealth.2022027)
Supplement: Supplementary file 1 [file publichealth-09-02-027-s001.pdf]

Public Health

AIMS Public Health, 9(2): 403–414.
DOI: 10.3934/publichealth.2022027
Received: 18 December 2021
Revised: 11 March 2022
Accepted: 20 March 2022
Published: 28 March 2022

http://www.aimspress.com/journal/aimsph

Research article

Evaluation of call volume and negative emotions in emergency response system telecommunicators: a prospective, intensive longitudinal investigation

Matthew Hoang[1], Elizabeth Hillier[2], Chris Conger[3], Devan N. Gengler[2], Cody W. Welty[2], Candace Mayer[2] and Patricia L. Haynes[2]*

[1] Department of Physiology, College of Medicine, University of Arizona, Tucson, AZ, USA
[2] Health Promotion Sciences, Mel and Enid Zuckerman College of Public Health, University of Arizona, Tucson, AZ, USA
[3] Tucson Fire Department, City of Tucson, Tucson, AZ, USA

* **Correspondence:** Email: thaynes@arizona.edu; Tel: +5206261855.

Supplementary Tables

Supplementary Table 1. Within-person centered correlations of shift-level study variables.

	**Variable**	**1**	**2**	**3**	**4**	**5**	**6**	**7**	**8**	**9**	**10**	**11**	**12**	**13**
1	No. days on-shift													
2	Shift duration, hrs	0.02												
3	Night-shift	–0.07	–0.06											
4	No. CAD calls / hr	–0.06	0.28**	0.14*										
5	No. CAD calls / hr, acute	0.01	0.06	0.02	0.25**									
6	No. CAD calls / hr, chronic	–0.02	0.26**	–0.01	0.46**	0.10								
7	Irritated, pre-shift	0.10	0.02	0.10	0.05	–0.05	0.07							
8	Irritated, post-shift	–0.01	0.14*	0.01	0.06	–0.02	–0.02	0.04						
9	Stressed, pre-shift	0.14*	0.03	0.01	0.10	–0.05	0.09	0.46**	–0.03					
10	Stressed, post-shift	–0.03	0.16**	0.01	–0.01	–0.06	–0.01	0.05	0.65**	0.04				
11	Worried, pre-shift	0.18**	0.00	0.07	0.02	–0.05	–0.02	0.41**	–0.04	0.65**	0.11			
12	Worried, post-shift	0.05	0.13*	0.01	–0.02	–0.04	–0.02	0.06	0.61**	0.14*	0.76**	0.15*		
13	Fatigued, pre-shift	0.21**	0.09	0.07	–0.08	–0.09	0.07	0.42**	0.07	0.27**	0.09	0.29**	0.10	
14	Fatigued, post-shift	–0.03	0.12*	0.10	–0.07	0.00	0.03	0.08	0.40**	0.05	0.31**	0.08	0.26**	0.17**

*Note: CAD = Computer-Assisted Dispatch system. All emotion ratings were conducted by Visual Analogue Scale ranging from 0 to 100. Post-shift emotion ratings and night-shift (coded 1 if began shift 10pm-2am, otherwise coded 0) were not centered within person. n = 281 for all correlations, except those involving pre-shift emotion variables (n = 269). *p < 0.05. **p < 0.01.

Supplementary Table 2. Variance decomposition for within-person variables.

Variable	Within-person variance	Between-person variance	ICC
Irritable	334.00	298.65	0.47
Stressed	256.03	304.38	0.54
Worried	216.60	250.07	0.54
Fatigued	310.14	229.38	0.43

*Note: ICC = intraclass correlation coefficient (between-person/total variance). Estimates are based on the total Level 1 sample size (n = 281 cases)

AIMS Press

© 2022 the Author(s), licensee AIMS Press. This is an open access article distributed under the terms of the Creative Commons Attribution License (http://creativecommons.org/licenses/by/4)
